# Genome-Wide Analysis and the Expression Pattern of the ERF Gene Family in *Hypericum perforatum*

**DOI:** 10.3390/plants10010133

**Published:** 2021-01-11

**Authors:** Qian Zhang, Wen Zhou, Bin Li, Lin Li, Meng Fu, Li Zhou, Xiaoding Yu, Donghao Wang, Zhezhi Wang

**Affiliations:** National Engineering Laboratory for Resource Development of Endangered Crude Drugs in Northwest China, Key Laboratory of the Ministry of Education for Medicinal Resources and Natural Pharmaceutical Chemistry, College of Life Sciences, Shaanxi Normal University, Xi’an 710119, China; zq182568@snnu.edu.cn (Q.Z.); wenzhou0229@snnu.edu.cn (W.Z.); libin1989@snnu.edu.cn (B.L.); shidalilin@snnu.edu.cn (L.L.); mengfu@snnu.edu.cn (M.F.); zhoulili@snnu.edu.cn (L.Z.); yuxiaoding1996@snnu.edu.cn (X.Y.); wangdonghao@snnu.edu.cn (D.W.)

**Keywords:** *Hypericum perforatum*, *ERF* gene family, expression pattern, qPCR

## Abstract

*Hypericum perforatum* is a well-known medicinal herb currently used as a remedy for depression as it contains many high levels of secondary metabolites. The ethylene response factor (ERF) family encodes transcriptional regulators with multiple functions that play a vital role in the diverse developmental and physiological processes of plants, which can protect plants from various stresses by regulating the expression of genes. Although the function of several *ERF* genes from other plants has been further confirmed, *H. perforatum* is the first sequenced species in Malpighiales, and no information regarding the *ERFs* has been reported thus far. In this study, a total of 101 *ERF* genes were identified from *H. perforatum*. A systematic and thorough bioinformatic analysis of the ERF family was performed using the genomic database of *H. perforatum*. According to the phylogenetic tree analysis, *HpERFs* were further classified into 11 subfamilies. Gene ontology (GO) analysis suggested that most of the *HpERFs* likely participate in the biological processes of plants. The cis-elements were mainly divided into five categories, associated with the regulation of gene transcription, response to various stresses, and plant development. Further analysis of the expression patterns showed that the stress-responsive *HpERFs* responded to different treatments. This work systematically analyzed *HpERFs* using the genome sequences of *H. perforatum*. Our results provide a theoretical basis for further investigation of the function of stress-related *ERFs* in *H. perforatum*.

## 1. Introduction

*Hypericum perforatum* is a perennial indigenous herb of the *Hypericum* genus that contains multiple medical compositions [1,2]. Many pharmaceutical companies have a keen interest in this plant due to its medicinal properties, in particular the presence of valuable secondary metabolites that are beneficial for the treatment of mild to moderate depression, cancer, and viral diseases [3,4,5]. Naphthodianthrones and phloroglucinols are the two primary biological active ingredients in *H. perforatum* [6,7]. Many kinds of transcription factor families could control the expression of various key enzyme genes that are related to the biosynthetic pathways of the main secondary metabolites, which indirectly affect the contents in plants.

Various abiotic stresses and hormone treatments could seriously regulate the process of growth and development in plants [8,9]. To cope with the environmental changes associated with abiotic stresses, a variety of signaling pathways are activated or inhibited in plants. The expression of stress-related genes is related to the coordinated regulation of diverse metabolic pathways, which is essential for plants to respond to various stresses [10]. This can affect the signal network to regulate the relative metabolic pathways and signal transduction [11].

The AP2/ERF superfamily plays a significant role in the responses to various stimuli by regulating the gene expression in plants. This superfamily was split into four subfamilies, which are named AP2, ERF, RAV, and Soloist [12]. All of the ERFs contain an AP2/ERF domain. This domain has a unique structure, which consists of three-stranded β-sheets and an α-helix, including about sixty conserved amino acids [13,14]. Not all of the ERF transcription factors regulate the same type of genes because the promoter region of each *ERF* gene is different and can bind diverse genes. Based on this, the ERF family was classified into the ERF and DREB subfamilies [15].

Studies have reported that the proteins from DREB or ERF subfamily both contained two highly conserved amino acids, which are the 14th valine (V14) and 19th glutamic acid (E19) or 14th alanine (A14) and 19th aspartic acid (D19) in their AP2/ERF domains, respectively. All of these conserved amino acids located in the structure of the β-sheet might play a significant role in specific binding to DNA sequences at specific sites [15]. At present, many ERFs have been verified to have various functions, including stress responses and the regulation of growth and development in plants, which are related to the ERF proteins located downstream of the ethylene signaling pathway [16,17,18,19].

The ERF proteins have been well investigated in many model plants, including *Arabidopsis thaliana* [20], rice [20], soybean [8], and maize [21]. Currently, there are 21,129 ERF sequences in the database of plant transcription factors (http://planttfdb.cbi.edu.cn/). Numerous studies manifested that the ERF family has vital roles in responding to stresses. For example, *AtERF4* can respond to abscisic acid (ABA), ethylene (ET), and jasmonic acid (JA) treatments [22]; the expression of *GmERF3* could be induced under ABA, salicylic acid (SA), and drought stresses [23]; and the *OsERF3* was found to be involved in ABA, drought, and salt treatments [24].

Although the research of *ERF* genes in many model plants gave us a better understanding of the ERF family, no information on this family was reported for *H. perforatum*. In this work, we performed a comprehensive genome-wide and expression pattern analysis of the *H. perforatum* ERF family under various abiotic stresses, including the cis-elements, gene ontology, gene structure, motif compositions, and expression patterns. Based on the sequence alignments and phylogenetic tree analysis, we divided the *HpERFs* into 11 subfamilies. The expression patterns of the *HpERFs* confirmed that the stress-responsive *HpERF* genes responded to various treatments. Our findings may provide a basis for selecting *ERF* genes for further functional investigations in *H. perforatum*.

## 2. Results

### 2.1. Identification of HpERFs and Their Sequence Features

A total of 101 presumed *ERF* genes were searched from the *H. perforatum* genomic databases using the BLASTP program with 122 AtERF proteins as queries. All *H. perforatum* ERF proteins (named HpERF1 to HpERF101) were confirmed to contain a single AP2/ERF domain by InterProscan. The basic features of the HpERFs were predicted, including the molecular weight (MW)/length of protein, isoelectric point (PI), and subcellular localization. Among the HpERFs, HpERF14 contained 33 amino acids, which was the smallest protein, while HpERF22 was the largest protein, containing 147 amino acids (Table S1).

The MW of the HpERFs ranged from 11.23512 to 49.28125 kDa. The pI of the HpERFs ranged from 4.3 (HpERF88) to 11.79 (HpERF14). The prediction of subcellular localization showed that 85 HpERF proteins lie in the nuclear region, twelve HpERF proteins were involved in the chloroplast region, two HpERF proteins (HpERF15 and HpERF40) were located in the mitochondrial region, only one HpERF protein (HpERF93) was located in the cytoplasmic region, and one HpERF protein (HpERF53) was involved in the extracellular region (Table S1).

The number of *HpERF* genes is less than the identified *ERF* genes in *Arabidopsis* (122) and rice (139) [20], while the *HpERF* family encodes more transcriptional regulators than *Salix* (88) [25] and *Prunus mume* (90) [26].

### 2.2. Analysis of Multiple Sequence Alignments and Cis-Acting Elements

**Figure 1:**
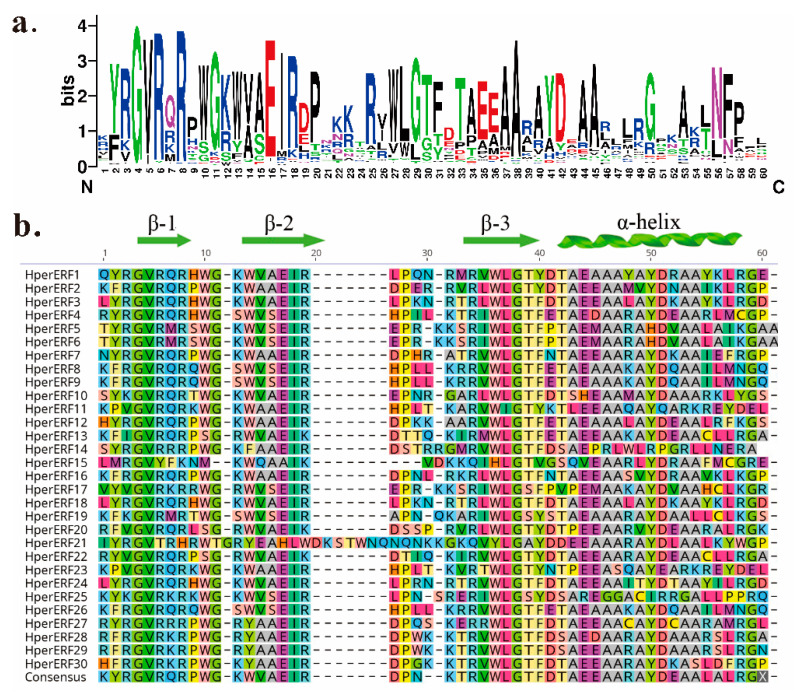
The sequence logo and multiple alignment analysis of the AP2/ERF domain. (**a**) The sequence logo of the AP2/ERF domain is displayed. (**b**) Multiple alignment analysis of the AP2/ERF domains was performed. The shading in different shapes and colors indicate the same and conserved amino acid residues, respectively. The green arrows and helix display the α-helix and β-sheet regions of the AP2/ERF domain, respectively.

The multiple sequence alignments of HpERFs in their conserved AP2/ERF domains were analyzed (Figure 1). The results revealed some highly conserved amino acids among all members of this family, including the 4th glycine (G), 6th arginine (R), 16th glutamic acid (E), 27th tryptophane (W), 28th leucine (L), 29th glycine (G), and 38th alanine (A) (Figure 1a). Almost all HpERFs had a conserved WLG motif (Figure 1b, Figure S1). Most of the HpERFs contained two crucial amino acid residues, which were the 14th alanine (A) and the 19th aspartate (D), and they are connected with the binding activity to the GCC-box in many ERFs [27,28]. The unique secondary structure of the AP2/ERF domain was also predicted to contain an α-helix and three β-sheets twined together in the opposite parallel direction, which were named β-1, β-2, and β-3 (Figure 1b, Figure S2). Compared with the other parts of this domain, the three β-sheets showed a slight structural fluctuation, while the α-helix and terminal regions presented a larger fluctuation.

**Figure 2:**
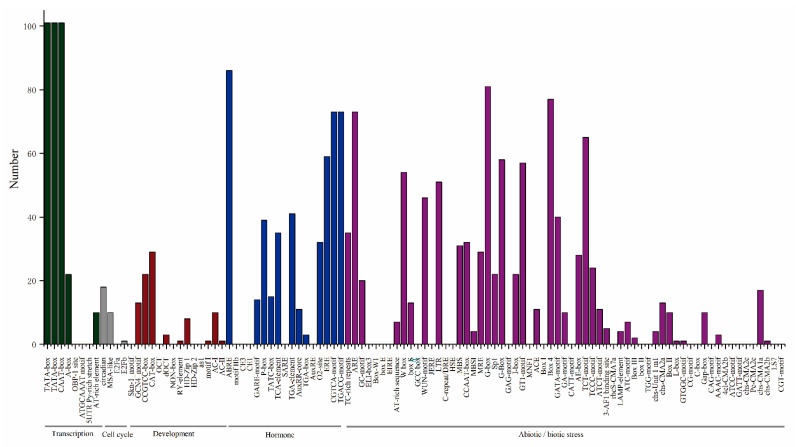
The distribution of various cis-acting elements in the promoter regions of *Hypericum perforatum ERF* genes (*HpERFs*). The cis-elements of *HpERFs* were searched using the online PlantCARE website. A graph is displayed according to the detected cis-elements relevant to specific elicitors/conditions/processes (x-axis) in the *HpERF* gene family members (y-axis).

To deeply research the function and regulatory roles of *HpERFs*, the various cis-elements of the promoter region were analyzed. As shown in Figure 2, the identified cis-acting elements were classified into five main functional classes: transcription, cell cycle, hormone, development, and abiotic or biotic stress. The statistics displayed that each of the *HpERFs* involved two elements: a TATA-box and CAAT-box, and most *HpERFs* contained at least one of the 26 presumed light-responsiveness elements.

In particular, 81 genes included a G-box element, 77 genes included a Box 4 element, and 65 members contained a light-responsive element (TCT-motif). The 51 genes also included a low-temperature-responsiveness element (LTR), 46 genes included a wound-responsive element (WUN-motif), 31 genes included drought-inducibility elements (MBS), and 35 genes had TC-rich repeat elements, which could protect plants from suffering abiotic and biotic stresses. There were 11 kinds of hormone-responsive elements found, which were related to the hormones: ABA, gibberellin (GA), SA, methyl jasmonate (MeJA), and ET (Table S2).

### 2.3. Phylogenetic Analysis of the ERF Family between H. perforatum and Arabidopsis

**Figure 3:**
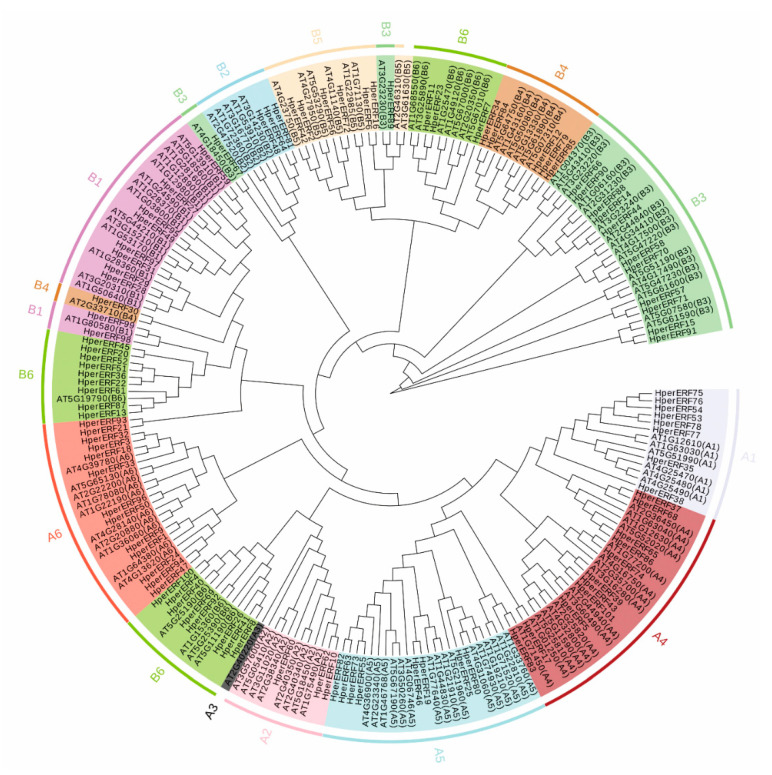
Phylogenetic tree of ethylene response factor (ERF) proteins from *H. perforatum* and *Arabidopsis*. The unrooted neighbor-joining (NJ) tree was constructed based on the amino acid sequences of ERFs from *H. perforatum* (101) and *Arabidopsis* (122) using MEGA6.06 with 1000 bootstrap replicates. The names of groups (A1–A6 and B1–B6) are shown outside of the circle, indicating different ERF subgroups.

To investigate the evolutionary relationships of ERF proteins between *H. perforatum* and *Arabidopsis*, all whole-length amino acid sequences of 101 HpERFs and 122 AtERFs were used to perform the analysis of protein multiple sequence alignment. An unrooted phylogenetic tree was built with the neighbor-joining (NJ) method, according to the similarity and topology of sequences, the 101 HpERFs were distributed into 11 subgroups (Figure 3). We regarded the classification of AtERFs as a reference and further named the 11 well-supported clades of HpERFs: A1 to A2, A4 to A6, and B1 to B6 [20].

The results could be very helpful to predict the various functions of unknown *HpERFs* according to the functionally confirmed in *AtERFs* or subgroups in *Arabidopsis*, which might contribute to the selection of target *HpERFs* for further functional analysis. For example, *HpERF88* might have the same function as *AtERF15,* which plays a key role in response to ABA and pathogen infection [29,30]. *AtERF73/HRE1* (subgroup B2) can be involved in regulating the ET response in both normoxia and hypoxia, which implies that *HpERF81*, *HpERF64,* and *HpERF48* might be related to the ethylene response in a negative regulatory role.

### 2.4. Gene Structure, Motif Composition, and Ka/Ks Analysis of HpERFs

**Figure 4:**
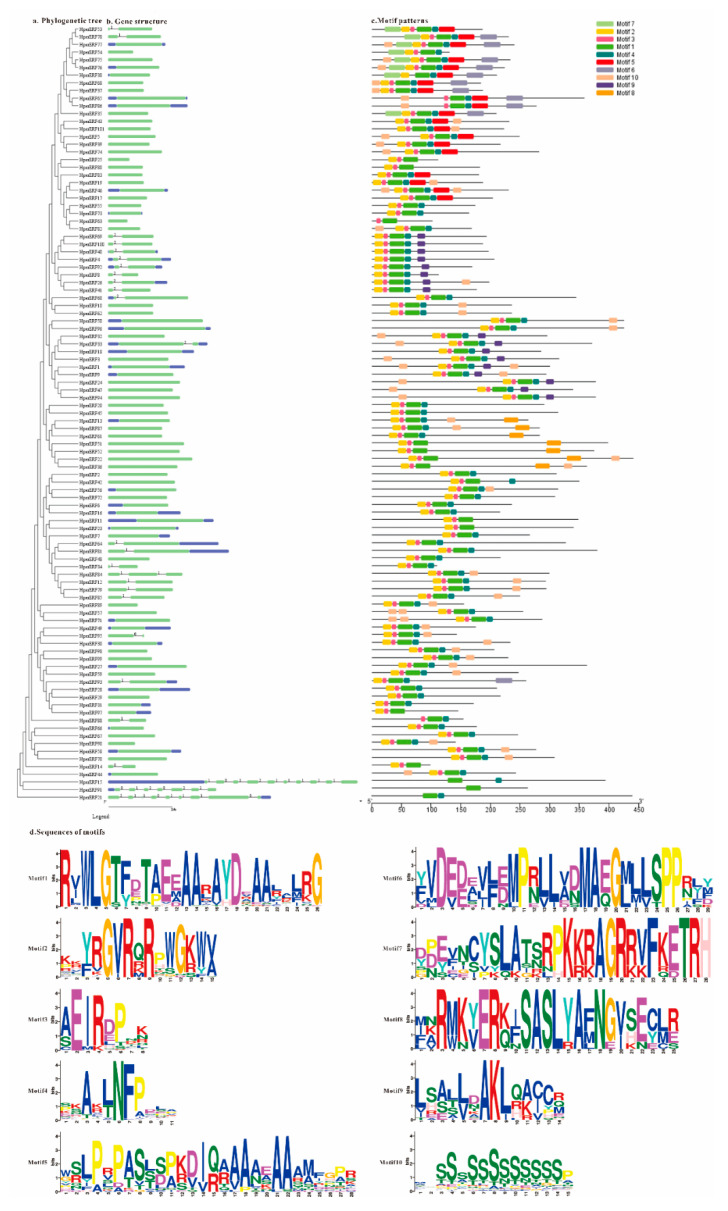
The phylogenetic relationships, gene structure, and composition of conserved motifs in ERFs from *H. perforatum*. (**a**) The phylogenetic tree on the left contains 101 ERF proteins (named HpERF1 to HpERF101) from *H. perforatum*. (**b**) Exon/intron structures of *ERF* genes from *H. perforatum*. Exon(s) and intron(s) are displayed by green boxes and black lines, respectively, while the untranslated regions are indicated by blue boxes. The number manifested the phases of according to the introns of *HpERFs*. (**c**) The motif patterns of 101 HpERF proteins. Each motif is showed by the box in different colors. (**d**) Sequence logos of each motif.

For insight into the composition of the structure and conserved motifs in HpERFs, we constructed a phylogenetic tree using the neighbor-joining (NJ) method based on the multiple sequence alignments of HpERFs (Figure 4a), and the genomic DNA sequences were used to analyze the intron and exon structures of *HpERFs* (Figure 4b). Structural analysis showed that 74.3% of the *HpERFs* contained only one exon without introns, while other exons of genes would be interrupted due to the existence of introns (Figure 4b). Typically, the majority of close *HpERFs* in the same cluster of the phylogenetic tree had a similar structure in terms of the intron number and exon length. A total of 10 conserved motifs were detected in the HpERFs (Figure 4c).

Each sequence logo of motifs is displayed (Figure 4d). All HpERFs contained Motif-1; about 90 genes contained Motif-2, Motif-3, and Motif-4; Motif-10 was involved in 56 genes; Motif-5, Motif-6, and Motif-9 existed in about 20 genes; Motif-7 only existed in nine genes, and only seven genes contained Motif-8 (Table S3). According to the results of the conserved motif analysis, we also found that close HpERFs in evolutionary relationships possessed the same motif compositions, which indicated the genes that belong to the same subgroups could have similar functions.

We found that there were 34 pairs of homologous genes in the *HpERFs* (Figure 4a), which suggests that about 67.3% *HpERFs* replicated and that the HpERF family has experienced gene expansion during the process of evolution. To research the influence of evolutionary factors on the HpERF family, the Ka/Ks ratios of 34 *HpERF* gene pairs were computed based on the phylogenetic tree (Figure 4). The results showed that the ratios of all of the orthologous gene pairs were no more than 1, which implies that a strong purifying selective pressure existed in all of these homologous genes during their evolution (Table S4).

### 2.5. Transcript Abundance Profiling by RNA-Seq

**Figure 5:**
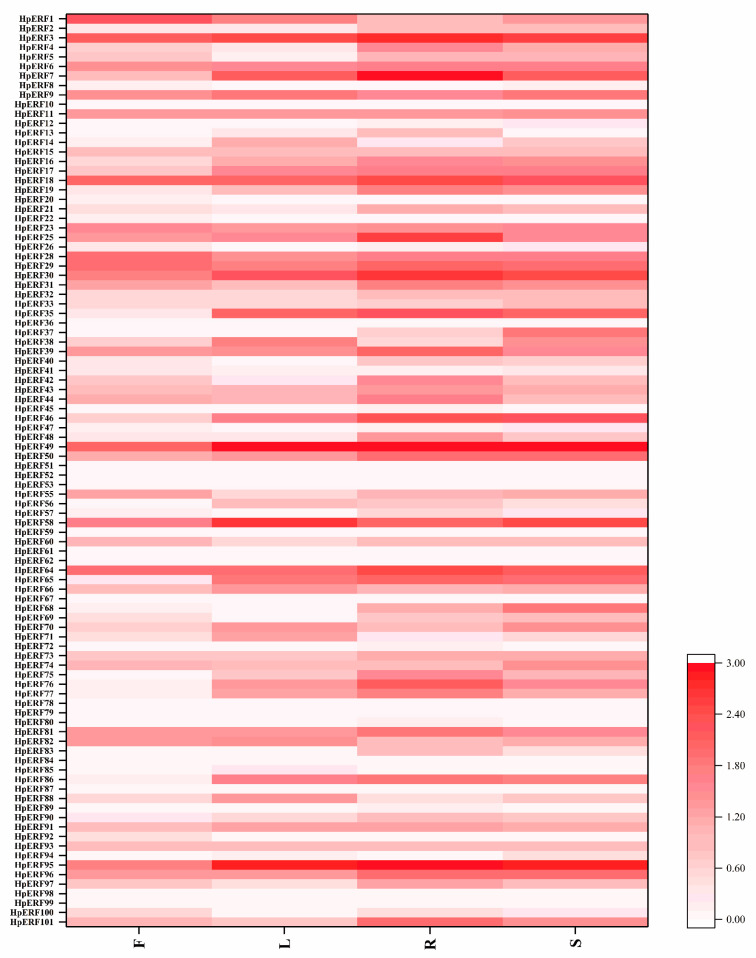
Heatmap showing the expression profile of *HpERFs* in four different tissues. The graph was generated based on log2-transformed count value from three replicates of RNA-Seq data using Origin software. Red and white boxes indicate high and low expression levels of *HpERFs*, respectively.

To explore the expression patterns of *HpERFs*, the transcriptome data of four organs from *H. perforatum* were used to analyze the transcript abundance (Figure 5). The sequencing results can be searched from the Sequence Read Archive of National Center for Biotechnology Information (SRA-NCBI) under the accession numbers SRR8438983 (flower), SRR8438984 (leaf), SRR8438985 (stem), and SRR8438986 (root). The transcripts of five *HpERFs* (*HpERF24*, *HpERF27*, *HpERF34*, *HpERF54,* and *HpERF63*) were not detected in the root, stem, leaf, or flower, which indicates that they might be pseudogenes. The expression levels of diverse *HpERFs* changed among the different tissues, among which 78 *HpERFs* were detected and expressed in four tissues (fragments per kilobase of exon model per million mapped reads (FPKM) > 0), and 29 *HpERFs* had relatively high expression levels across the four tissues (FPKM > 5), in particular *HpERF3*, *HpERF30*, *HpERF49*, *HpERF64,* and *HpERF95*.

As is shown in Figure 5, we also found that *HpERF95* showed the highest transcript accumulation in the root and stem, while *HpERF49* and *HpERF1* showed the highest transcript accumulation in the leaf and flower, respectively. Based on the phylogenetic tree analysis, *HpERF49* and *AtERF4*(*At3g15210*) were classified into the same subgroups, which manifested that the expression of *HpERF49* could be induced by ET, ABA, and JA [22]. We randomly chose five genes (*HpERF7, HpERF30, HpERF49, HpERF58,* and *HpERF95*) to examine the RNA-seq data by qPCR, which confirmed that the expression trend of these genes in four tissues was the same as the results of transcriptome sequencing (Figure S3).

### 2.6. Gene Ontology (GO) Annotation

**Figure 6:**
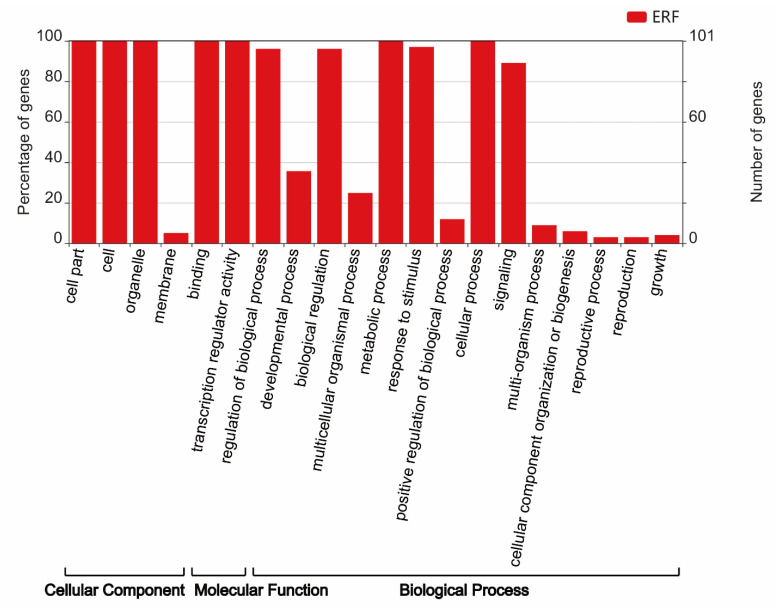
Functional classifications of *HpERFs* according to the gene ontology (GO) terms assigned to genes.

Gene ontology (GO) enrichment was analyzed including the biological process, molecular function, and cellular components, which is beneficial to understand the function of proteins from the molecular level. Therefore, GO enrichment analysis of *HpERFs* was further performed using Blast2GO software (Figure 6). Among the 20 different functional groups: four groups were identified that were relevant to cellular components, three groups were identified as involved in molecular functions, and the other thirteen groups could play a vital part in the biological processes of plants (Table S5). In *H. perforatum*, *ERF* genes were mostly classified into various GO categories (Figure 6), except for *HpERF24*, *HpERF34, HpERF54*, and *HpERF63*.

The results from the molecular functions GO term revealed that 72% of *HpERFs* had the function of DNA binding (GO: 0003677), which implies that these genes might regulate the transcription and expression of genes by DNA binding. The biological process GO term showed that 84 *HpERFs* could participate in the ethylene-activated signaling pathway (GO: 0009873), five *HpERFs* (*HpERF3, HpERF18, HpERF33, HpERF78,* and *HpERF84*) could be involved in the cold stimulus (GO: 0009409), and six genes might play a vital role in the response to salt stress (GO: 0009651). The cellular component GO term demonstrated that most of *HpERFs* (96%) were located in the nucleus (GO: 0005634), among which seven HpERFs were located in the nucleus and cytoplasm (GO: 0005737), and only *HpERF30, HpERF49,* and *HpERF95* were located in the nucleus and membrane (GO: 0016020).

### 2.7. Analysis of Expression Patterns under Different Stress Treatments

Depending on the phylogenetic distance with the known stress-related ERF proteins or subgroups in model plants and the results of cis-element analysis (Table S2), 10 *HpERFs* were chosen to further analyze the levels of their expression. Among them *HpERF27, HpERF31, HpERF49,* and *HpERF97* belonged to the B1 subgroup whose genes may be involved in various hormone stresses [22,31,32,33]; *HpERF48, HpERF64,* and *HpERF81* were clustered in the B2 subgroup, which might be related to salt and freezing tolerance [33,34,35,36]; *HpERF58* and *HpERF70* belonged to the B3 subgroup whose genes could be induced by drought, high salt, SA, ET, and JA [33,37,38,39]; and the *HpERF78* of the A1 subgroup might play a key role in the response to low-temperature, salt, and drought stresses [33,40,41,42].

**Figure 7:**
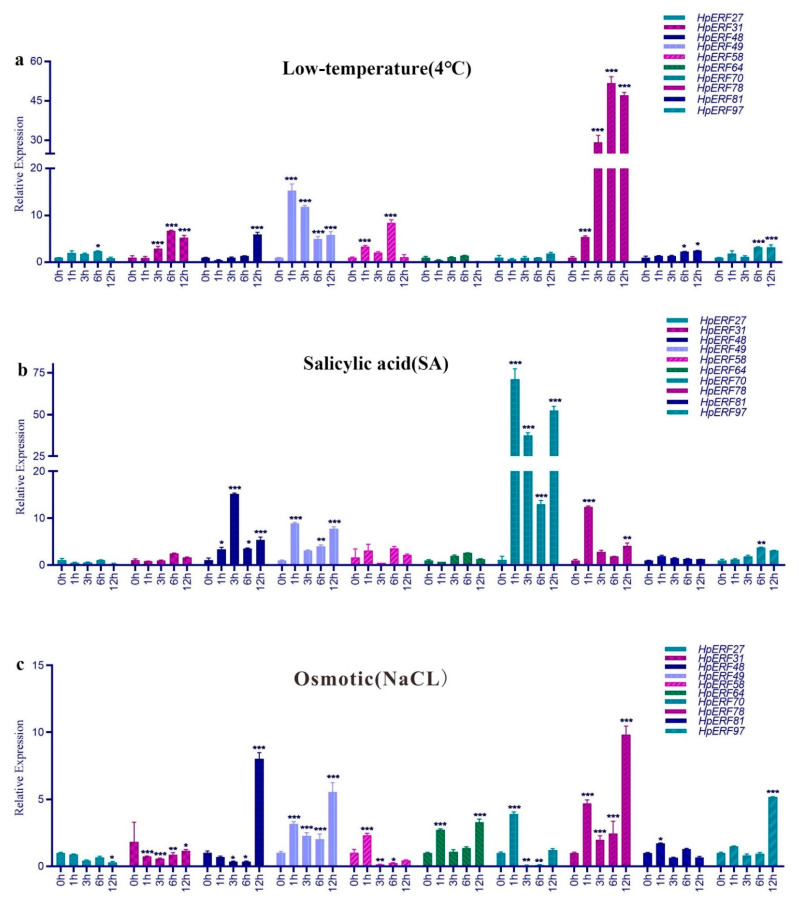
The expression levels of 10 *HpERFs* in response to various stresses treatments at 0, 1, 3, 6, and 12 h. (**a**) 4 ℃. (**b**) salicylic acid (SA). (**c**) osmotic. The data were normalized to the *HpACT2* gene and analyzed with a two-way method using GraphPad Prism, version 8. Asterisks (*p* < 0.05) indicate significant differences compared with the control group (* *p* < 0.05, ** *p* < 0.01, *** *p* < 0.001).

The expression levels of selected *HpERFs* were presented during the three stress conditions in Figure 7. Almost all genes were upregulated under low-temperature stress, except for *HpERF64* and *HpERF70,* which had no significant differences shown in the comparison. Among them, *HpERF49* and *HpERF78* were upregulated about 10-fold at 1 h and 50-fold at 6 and 12 h after treatment, respectively (Figure 7a). Nine genes were upregulated under SA stress, except for *HpERF27* (Figure 7b). Among them, *HpERF70* was mostly upregulated about 70-fold at 1 h, and two genes, *HpERF48* and *HpERF78,* were upregulated about 15-fold at 3 and 1 h after treatment, respectively.

Under osmotic stress (Figure 7c), *HpERF27* and *HpERF31* were downregulated, while the genes *HpERF49, HpERF64*, *HpERF78*, *HpERF81,* and *HpERF97* were upregulated, among which *HpERF78* was upregulated about 10-fold at 12 h after treatment. *HpERF48* was downregulated at 1 h and upregulated about 10-fold at 12 h after treatment, while both *HpERF58* and *HpERF70* were upregulated at 1 h and downregulated at 3 and 6 h after treatment. *HpERF49* and *HpERF97* were upregulated about 5-fold at 12 h after treatment.

## 3. Discussion

The ERF family is one of the largest families of plant transcription factors, which plays a vital role in the process of growth and development and regulating the response to biotic and abiotic stresses in plants [15,33,43]. Although this family has been widely researched in many plants, such as *Arabidopsis* [20], rice [20], *Vitis vinifera* [11], and peaches [43], the identification and functions of *ERFs* based on the whole-genome sequence of *H. perforatum* have not been studied so far. Therefore, a comprehensive and systematic analysis of the *ERF* gene family was carried out in *H. perforatum*.

In this study, a total of 101 *ERF* genes were identified in *H. perforatum*. According to previous studies, the number of *H. perforatum ERFs* was less than that in maize (107 genes) [21,44], *Arabidopsis* (122 genes) [20], rice (139 genes) [20], and poplar (209 genes) [45], which implies that the ERF family in these species has expanded in comparison with *H. perforatum* [46]. The number of various studied species is different, which may be caused by gene duplication, including tandem duplication and segmental duplication [47,48]. At present, studies also indicated that the expansion of the *ERF* gene family was related to the events of gene duplication in plants, which also showed that the primary driving force working on the evolution of this family was natural pressures [49,50].

To further excavate the restricted conditions in the evolution of the *HpERF* family, the Ka/Ks ratios of the 34 paralogous *HpERF* gene pairs were computed. The smaller the value of Ka/Ks is between gene pairs, the more severe the selective constraint they have evolved [50]. The Ka/Ks ratios of all duplicated *HpERF* gene pairs were no more than 1, which confirmed a strong purifying selective pressure existed in this family during its evolution (Table S4). This suggests that the *ERF* gene families are currently being purified under selection in *H. perforatum*. Based on the phylogenetic analysis of the *ERF* family between *H. perforatum* and *Arabidopsis thaliana*, the *HpERF* genes were classified into 11 subgroups, which also could provide a credible reference for predicting the function of target *HpERF* genes (Figure 3).

Some HpERFs were clustered in the same branch with the known AtERFs and subgroups, which showed that the conservation of ERF functions between the two species. For example, the HpERFs in A1 and B2 may play an important role in regulating the salt and freezing tolerance, while B1 and B3 could be involved in the response to various hormone stimuli, including ET, MJ, SA, and ABA according to the conservation of ERF functions [20,32,34,36]. Therefore, the *HpERFs* in the A1, B1, B2, and B3 subgroups should be regarded as key candidate genes for further research that involves a response to various abiotic stresses.

There were many studies of plant ERF transcription factors involved in plant growth and development mainly focused on *Arabidopsis thaliana*. For example, the over-expression of *RAP2.2* (*At3g14230*) and *RAP2.12* (*At1g53910*) was shown to improve the survival rate of plants in the hypoxia condition, while *ERF1* (*At3g23240*) was shown to mediate the ethylene responses after pathogen invasion as a negative regulator [51,52]. Therefore, the over-expression of *HpERF44* might reduce tolerance against the pathogen, and *HpERF48*, *HpERF64*, and *HpERF81* may have a similar function with *RAP2.2* and *RAP2.12* based on the phylogenetic tree.

The *HpERF89* and *AtERF98* (*At3g23230*) clustered in the same branch might regulate the expressions of the genes that are connected with the biosynthesis of ascorbic acid (AsA) [53]. The research reported that *AtERF15* (*At2g31230*) is nuclear-localized, is involved in the response to ABA, and mediates the seed development, germination, and seedling growth, which indicates that *HpERF88* might play similar roles in *H. perforatum* [29]. The multiple sequence alignment is one of the vital methods of researching bioinformatics, and it plays a key role in the evolution analysis and protein structure prediction [54].

The results of the sequence alignments revealed characteristic amino acids, such as the majority of the HpERFs containing two crucial amino acid residues, which were the 14th alanine (A) and the 19th aspartate (D), which are connected with the binding activity to the GCC-box in many ERFs (Figure 1a,b, Figure S1) [13,20,55]. The secondary structural elements of this domain were found to contain an α-helix and three β-sheets (Figure 1b, Figure S2). A study reported that these three β-sheets were closely related to the recognition of target DNA sequences, especially the highly conserved arginine and tryptophan residues, which play a critical role that is essential for *ERFs* to carry out their regulatory functions in plants [15].

Structural analysis determined that 74.3% of the *HpERFs* did not contain introns (Figure 4), and this was a representative characteristic of the *ERFs* [15,56]. The close *HpERFs* had a similar structure and conserved motifs, which also confirmed that the classification was credible. Conserved motifs might regulate the expression of specific genes as possible DNA binding sites. The transcriptional activity and DNA combination specificity were determined by the unique structures and motifs. Therefore, we predicted that the HpERFs with the same subgroups could have similar functions according to the gene structure and motif composition analysis.

The results of the cis-elements and GO analysis suggested that *HpERFs* had multiple functions in various biological processes (Figure 2 and Figure 6, Table S5). To deeply explore the information of *HpERFs’* functions, we analyzed the transcript abundance of *HpERFs* and found that most of the *HpERFs* were differentially expressed in four tissues (Figure 5). The expressions of genes were not detected in our libraries, which implied that they might be pseudogenes. Drought, cold, and phytohormones play vital roles in responding to abiotic stresses [57]. Many studies showed that *ERF* genes participate in multiple abiotic stress responses [15,32,53].

Therefore, for further study of the functions of *ERF* genes, we analyzed the expression levels of 10 selected *HpERFs* in various treatments (Figure 7). Nearly all of the genes were upregulated after low-temperature treatment, except for *HpERF64* and *HpERF70,* which had no significant differences in the comparison, suggesting the eight selected genes were involved in the response to this stress and may regulate the transcriptions of related genes. Among them, *HpERF49* and *HpERF78* were upregulated about 10-fold at 1 h and 50-fold at 6 and 12 h after treatment, respectively (Figure 7a). Nine genes were upregulated under SA stress, but not *HpERF27* (Figure 7b). Among them, *HpERF70* was mostly upregulated about 70-fold at 1 h and *HpERF48* and *HpERF78* were upregulated about 15-fold at 3 and 1 h after treatment, respectively.

The results showed that *HpERF48*, *HpERF70*, and *HpERF78* likely participate in the SA response. Under osmotic stress (Figure 7c), *HpERF27* and *HpERF31* were downregulated, while the genes *HpERF49, HpERF64*, *HpERF78*, *HpERF81,* and *HpERF97* were upregulated, among which *HpERF78* and *HpERF48*, *HpERF49,* and *HpERF97* were separately upregulated about 10-fold and 5-fold at 12 h after treatment, which indicated that these genes may participate in the signaling pathways of osmolyte synthesis [58,59].

According to the phylogeny analysis, the selected *HpERF* genes exhibited a similar stress-responsive expression pattern to the model species in the same clade or subgroup. Among them, *HpERF48* (B2), *HpERF49* (B1), and *HpERF78* (A1) were upregulated in three treatments, in particular, *HpERF78* was upregulated about 50-fold during low-temperature stress, *HpERF70*(B3) was upregulated about 70-fold during SA stress. Therefore, we predicted that *HpERF48*, *HpERF49*, *HpERF70,* and *HpERF78* were vital in responding to low-temperature, SA, and drought stresses. These four genes can be used as candidate genes for later functional research. In brief, the results of our study could give a credible reference for further research of the functions of *ERF* genes in *H. perforatum*.

## 4. Materials and Methods

### 4.1. Plant Material and Treatment

Seeds (2n = 2x = 16) of *H. perforatum* were collected from the Qinling Mountains and were sprouted on a seedling bed in a greenhouse at 25 °C with natural lighting (16 h light/8 h dark) and 60–80% humidity after sterilizing for 6 minutes and washing 10 times with 10% sodium hypochlorite and sterile water, respectively. For the hormonal treatment, the two-month-old seedlings were subjected to different treatments for 0 (as control samples), 1, 3, 6, and 12 h in MS solid medium by spraying the seedlings with 10 mM salicylic acid (SA). For the low-temperature (4 °C) stress treatment, plants were planted in a pot with MS under 4 °C condition. For the osmotic stress treatment, plants were placed in a solution with 200 mM NaCl. The stress samples were taken respectively at 0, 1, 3, 6, and 12 h with three biological replicates. Each sample was frozen instantly in liquid nitrogen and stored at −80 °C for further RNA isolation.

### 4.2. Database Search for Sequences and the Prediction of the Subcellular Locations

The completed genome sequences of the ERF proteins in *H. perforatum* were detected and assembled by our laboratory (Table S6) [60]. To further confirm all of the proteins belonging to ERF family, their sequences were used to search a single AP2/ERF domain by Interpro (http://www.ebi.ac.uk/interpro/). The results were that 101 ERF proteins were identified in *H. perforatum*. The ERF protein sequences of *Arabidopsis thaliana* were downloaded from the Tair database (https://www.arabidopsis.org/). The basic information of *H. perforatum* ERFs was acquired using the ExPasy website (http://web.expasy.org/protparam/). CELLO v.2.5 (http://cello.life.nctu.edu.tw/) was used to predict the subcellular locations of the HpERFs.

### 4.3. Multiple Alignments and Phylogenetic Tree Analysis

For multiple alignment analysis, the SMART program (http://smart.embl.de/) was used to acquire the core sequence of the AP2/ERF domain, and the core sequences of the HpERFs were further analyzed by the Geneious v 9.1.4 and ClustalX v 2.1 software programs. The online program of Weblogo (http://weblog.berkeley.edu/logo.cgi) was used to show the characteristics of the domain. For the phylogenetic tree analysis of the HpERFs, the neighbor-joining (NJ) method was used to construct a phylogenetic tree using MEGA v 6.0 with 1000 bootstraps. Another phylogenetic tree was built between *H. perforatum* and *Arabidopsis* using the same method.

### 4.4. The Prediction of Conserved Motifs and Gene Structure Analysis

The online MEME (http://meme-suite.org/tools/meme) program was utilized to search and detect protein motifs of 101 HpERFs with expected e-values less than 2 × 10^−30^ [61]. The following parameter settings default changed the number of motifs into 10. Then, the result of the XML file obtained from MEME was displayed with TBtools v 0.58 [62]. The exon/intron structures of the *HpERFs* were displayed using GSDS (http://gsds.cbi.pku.edu.cn) with the genomic DNA sequences [63].

### 4.5. Cis-Elements and Ka/Ks Analysis

The 1500 bp promoter sequences located upstream of the gene start codon were obtained from the whole-genome sequences of *H. perforatum* using the BioEdit software. The potential cis-elements of *HpERFs* were searched in the PlantCARE database [64]. With the purpose of examining whether positive selection existed in the evolution of *HpERF* genes, the online websites of Clustal Omega (https://www.ebi.ac.uk/Tools/msa/clustalo/) and PAL2NAL (http://www.bork.embl.de/pal2nal/) were used to calculate the synonymous substitution rate (Ks) and nonsynonymous substitution rate (Ka) values of homologous gene pairs with their amino acid sequences in *HpERFs* [65,66].

### 4.6. Gene Ontology Annotation Analysis

The GO annotations of *HpERFs* were analyzed using the Blast2GO software and displayed by the online website BGI WEGO (https://biodb.swu.edu.cn/cgi-bin/wego/index.pl) [67,68]. Then, the GO terms of genes were acquired using BLASTP. Subsequently, the various functions of genes were further classified and distributed with the AmiGO website (http://amigo1.geneontology.org/cgi-bin/amigo/term_details) [69].

### 4.7. Isolation of RNA and cDNA Preparation

The Polysaccharide and Polyphenols Plant Quick RNA Isolation Kit (Waryong, Beijing, China) was used to isolate and extract the total RNA of the samples treated with diverse stresses according to the manufacturer’s instructions. The elimination of the genomic DNA in the samples was carried out using a mixture of RNase-free DNase I and buffer (Waryong, Beijing, China) for approximately 20 to 30 minutes in the adsorption column at room temperature, and then the RNase-free DNase I stayed on the adsorption column through centrifugation.

The quality of RNA was detected using 1% (p/v) gel electrophoresis, and a NanoDrop 2000c spectrophotometer (Thermo Scientific, MA, USA) was utilized to measure the concentration and ratios (A260/A280 and A260/A230) of the RNA. For cDNA synthesis, following the instructions of the PrimeScriptTM RT Reagent Kit (TaKaRa, China), we used a total of 1 μg RNA for reverse-transcription in a 20 µL volume. The qPCR primers were designed according to the *HpERF* sequences using the online website (https://www.genscript.com/tools/real-time-pcr-taqman-primer-design-tool), and we further detected the primer specificity to certify that the target gene fragments could be accurately amplified.

### 4.8. RNA-Seq Expression and qPCR Analysis

The four different tissues (root, stem, leaf, and flower) of two-year-old *H. perforatum* were collected individually in the flowering stage of development. These samples were used to perform the transcriptome sequencing and to further detect the tissue-specific expression patterns of the *HpERFs*. The RNA-Seq data can be obtained from the Sequence Read Archive (SRA) database of NCBI with the accession numbers SRR8438983 (flower), SRR8438984 (leaf), SRR8438985 (stem), and SRR8438986 (root) [70,71]. The method of computing the transcript abundance of *HpERFs* was following the fragments per kilobase of exon model per million mapped reads (FPKM).

Real-time fluorescent quantitative PCR (qPCR) was carried out using a Roche LightCycler 96 system (Roche Diagnostics GmbH) with ChamQTM SYBR^®^ qPCR Master Mix (Vazyme, Nanjing, China). The PCR conditions were set as 95 °C for 30 s, 95 °C for 5 s, and 60 °C for 30 s with 45 cycles. Each reaction had three biological and technical replicates and used 30-fold diluted cDNA as a template. The 2^−^^△△CT^ method was used to calculate the corresponding expression values of *HpERFs*. According to the studies of our laboratory, *HpACT2* (MK054303) and *HpTUB-β* (MK106361) were selected as top two stable and reliable reference genes, especially *HpACT2* proved extremely stable in different treatments [70,71]. Furthermore, the results of our preliminary experiment revealed that the expressions using *HpTUB-β* and *HpACT2* as the internal references had no significant difference with those obtained using *HpACT2*. Therefore, the *HpACT2* gene was selected as the internal reference gene in this work, and the reference genome data can be searched from NCBI in SRA under the BioProject number PRJNA588586 [70]. The primer sequences are listed in Table S7.

## 5. Conclusions

In summary, a first comprehensive analysis of the *HpERF* gene family regarding the phylogenetic relationships, gene structures, conserved motifs, cis-acting elements, gene ontology, and expression profiles of *HpERFs* was performed in this work, which indicated that *HpERFs* have important roles in response to low-temperature, SA, and osmotic stresses. Based on the analysis of the phylogenetic tree and expression patterns, we predicted and verified the possible functions of the *HpERFs* in our study. Our findings provide a credible reference for further exploring the regulation mechanisms and stress resistance in the growth and development of *H. perforatum*.

## Data Availability

The raw data of the transcriptome analysis used in this study was submitted to the Sequence Read Archive (SRA) at NCBI under accession numbers SRR8438983 (flower), SRR8438984 (leaf), SRR8438985 (stem), and SRR8438986 (root). The reference genome data are deposited in SRA under project number PRJNA588586.

## Supplementary Materials

The following are available online at https://www.mdpi.com/2223-7747/10/1/133/s1, Table S1: The predicted features of the 101 HpERFs, Table S2: The promoter cis-elements analysis of the *ERF* genes in *H. perforatum*, Table S3: Analysis and distribution of conserved motifs of the 101 HpERF proteins, Table S4: The Ka/Ks calculation of the duplicated *ERF* gene pairs in *H. perforatum*, Table S5: Functional classifications of *HpERFs* according to the GO terms assigned to genes, Table S6: List of the 101 *HpERF* genes identified in this study, Table S7: The primers used in this study, Figure S1: The multiple alignment analysis of the AP2/ERF domains from the 101 *H. perforatum* ERF proteins, Figure S2: The 3D structure of the AP2/ERF domain. The secondary structural elements of this domain, including α-helix and β-sheet, are labeled and the ends are N-terminal and C-terminal, respectively, Figure S3: The expression patterns of five *HpERFs* in the root (R), stem (S), leaf (L), and flower (F) tissues examined by qPCR. Asterisks (*p* < 0.05) indicate significant differences compared with the control (* *p* < 0.05, ** *p* < 0.01, *** *p* < 0.001).

## References

[1] Sirvent T.M., Krasnoff S.B., Gibson D.M. (2003). Induction of Hypericins and Hyperforins in Hypericum perforatum in Response to Damage by Herbivores. J. Chem. Ecol..

[2] Weina H., Preeti S., Gregory F. (2016). A Perspective on Hypericum perforatum Genetic Transformation. Front. Plant Sci..

[3] Zdunić G., Gođevac D., Milenković M., Šavikin K., Menković N., Petrović S. (2010). Anti-inflammatory and Gastroprotective Properties of Hypericum richeri Oil Extracts. Nat. Prod. Commun..

[4] Palo J. (1999). Herbal Medicines: The Complete German Commission E Monographs: Therapeutic Guide to Herbal Medicines. Am. Med. Assoc..

[5] Butterweck V. (2003). Mechanism of Action of St John’s Wort in Depression: What is Known?. CNS Drugs.

[6] Butterweck V., Wall A., Liefländer-Wulf U., Winterhoff H., Nahrstedt A. (1997). Effects of the total extract and fractions of Hypericum perforatum in animal assays for antidepressant activity. Pharmacopsychiatry.

[7] Group H.D.T.S., Davidson J.R.T., Gadde K.M., Fairbank J.A., Weisler R.H. (2002). Effect of Hypericum perforatum (St John’s Wort) in Major Depressive Disorder: A Randomized Controlled Trial. JAMA.

[8] Zhang G., Chen M., Chen X., Xu Z., Guan S., Li L.C., Li A., Guo J., Mao L., Ma Y. (2008). Phylogeny, gene structures, and expression patterns of the ERF gene family in soybean (*Glycine max* L.). J. Exp. Bot..

[9] Chinnusamy V., Zhu J., Zhu J.K. (2007). Cold stress regulation of gene expression in plants. Trends Plant Sci..

[10] Ito T.M., Polido P.B., Rampim M.C., Kaschuk G., Souza S.G.H. (2014). Genome-wide identification and phylogenetic analysis of the AP2/ERF gene superfamily in sweet orange (Citrus sinensis). Genet. Mol. Res..

[11] Zhuang J., Peng R.H., Cheng Z.M., Zhang J., Cai B., Zhang Z., Gao F., Zhu B., Fu X.Y., Jin X.F. (2009). Genome-wide analysis of the putative AP2/ERF family genes in Vitis vinifera. Entia Hortic..

[12] Ohme-Takagi M., Shinshi H. (1995). Ethylene-Inducible DNA Binding Proteins That Interact with an Ethylene-Responsive Element. Plant Cell.

[13] Allen M.D., Yamasaki K., Ohme-Takagi M., Tateno M., Suzuki M. (1998). A novel mode of DNA recognition by a beta-sheet revealed by the solution structure of the GCC-box binding domain in complex with DNA. EMBO J..

[14] Shi Q., Dong Y., Qiao D., Wang Q., Ma Z., Zhang F., Zhou Q., Xu H., Deng F., Li Y. (2015). Isolation and characterization of ZmERF1encoding ethylene responsive factor-like protein 1 in popcorn (Zea mays L.). Plant Cell Tissue Org..

[15] Sakuma Y., Liu Q., Dubouzet J.G., Abe H., Shinozaki K., Yamaguchi-Shinozaki K. (2002). DNA-binding specificity of the ERF/AP2 domain of Arabidopsis DREBs, transcription factors involved in dehydration- and cold-inducible gene expression. Biochem. Biophys. Res. Commun..

[16] Banno H., Ikeda Y., Niu Q.W., Chua N.H. (2001). Overexpression of Arabidopsis ESR1 Induces Initiation of Shoot Regeneration. Plant Cell.

[17] Deng B., Huang Z.J., Ge F., Liu D.Q., Lu R.J., Chen C.Y. (2017). An AP2/ERF Family Transcription Factor PnERF1 Raised the Biosynthesis of Saponins in Panax notoginseng. J. Plant Growth Regul..

[18] Yu Z.X., Li J.X., Yang C.Q., Hu W.L., Wang L.J., Chen X.Y. (2012). The Jasmonate-Responsive AP2/ERF Transcription Factors AaERF1 and AaERF2 Positively Regulate Artemisinin Biosynthesis in *Artemisia annua* L.. Mol. Plant.

[19] Stockinger E.J., Gilmour S.J., Thomashow M.F. (1997). Arabidopsis thaliana CBF1 encodes an AP2 domain-containing transcriptional activator that binds to the C-repeat/DRE, a cis-acting DNA regulatory element that stimulates transcription in response to low temperature and water deficit. Proc. Natl. Acad. Sci. USA.

[20] Nakano T., Suzuki K., Fujimura T., Shinshi H. (2006). Genome-Wide Analysis of the ERF Gene Family in *Arabidopsis* and Rice. Plant Physiol..

[21] Du H.W., Huang M., Zhang Z.X., Cheng S.Y. (2014). Genome-wide analysis of the AP2/ERF gene family in maize waterlogging stress response. Euphytica.

[22] Yang Z., Tian L., Latoszek-Green M., Brown D., Wu K. (2005). Arabidopsis ERF4 is a transcriptional repressor capable of modulating ethylene and abscisic acid responses. Plant Mol. Biol..

[23] Zhang G., Chen M., Li L., Xu Z., Chen X., Guo J., Ma Y. (2009). Overexpression of the soybean GmERF3 gene, an AP2/ERF type transcription factor for increased tolerances to salt, drought, and diseases in transgenic tobacco. J. Exp. Bot..

[24] Zhang H., Zhang J., Quan R., Pan X., Wan L., Huang R. (2013). EAR motif mutation of rice OsERF3 alters the regulation of ethylene biosynthesis and drought tolerance. Planta.

[25] Guodong R., Jinkai S., Yanfei Z., He C., Zhang J. (2015). Genome-wide analysis of the AP2/ERF gene family in Salix arbutifolia. FEBS Open Bio.

[26] Du D.L., Hao R.J., Cheng T.R., Pan H.T., Yang W.R., Wang J., Zhang Q.X. (2013). Genome-Wide Analysis of the AP2/ERF Gene Family in Prunus mume. Plant Mol. Biol. Rep..

[27] Wang S., Yang S., Yin Y., Xi J., Li S., Hao D. (2010). Molecular dynamics simulations reveal the disparity in specific recognition of GCC-box by AtERFs transcription factors super family in Arabidopsis. J. Mol. Recognit..

[28] Wang L., Qin L., Liu W., Zhang D., Wang Y. (2014). A novel ethylene-responsive factor from Tamarix hispida, ThERF1, is a GCC-box- and DRE-motif binding protein that negatively modulates abiotic stress tolerance in Arabidopsis. Physiol. Plant.

[29] Lee S.B., Lee S.J., Kim S.Y. (2015). AtERF15 is a positive regulator of ABA response. Plant Cell Rep..

[30] Zhang H., Huang L., Dai Y., Liu S., Hong Y., Tian L., Huang L., Cao Z., Li D., Song F. (2015). Arabidopsis AtERF15 positively regulates immunity against Pseudomonas syringae pv. tomato DC3000 and Botrytis cinerea. Front. Plant Sci..

[31] Li Z., Zhang L., Yu Y., Quan R., Zhang Z., Zhang H., Huang R. (2011). The ethylene response factor AtERF11 that is transcriptionally modulated by the bZIP transcription factor HY5 is a crucial repressor for ethylene biosynthesis in Arabidopsis. Plant J..

[32] Wei L., Umuhoza K.N.J., Wu T., Yang Y., Zhang X., Xu X., Yi W., Han Z., Wu K. (2017). The ethylene response factor AtERF4 negatively regulates the iron deficiency response in Arabidopsis thaliana. PLoS ONE.

[33] Lee J.H., Hong J.P., Oh S.K., Lee S., Choi D., Kim W. (2004). The ethylene-responsive factor like protein 1 (CaERFLP1) of hot pepper (Capsicum annuum L.) interacts in vitro with both GCC and DRE/CRT sequences with different binding affinities: Possible biological roles of CaERFLP1 in response to pathogen infection and high salinity conditions in transgenic tobacco plants. Plant Mol. Biol..

[34] Yi S.Y., Kim J.H., Joung Y.H., Lee S., Kim W.T., Yu S.H., Choi D. (2004). The pepper transcription factor CaPF1 confers pathogen and freezing tolerance in Arabidopsis. Plant Physiol..

[35] Yang Y.H., Yin L.R., Hong G.E., Huang R.F., Xiao Q.M., Xie B.Y. (2007). Overexpression of a Novel Member of ERF Transcription Factor JERF3 in Lily Enhances the Tolerance to Salt. Acta Hortic. Sin..

[36] Gao S., Zhang H., Tian Y., Li F., Zhang Z., Lu X., Chen X., Huang R. (2008). Expression of TERF1 in rice regulates expression of stress-responsive genes and enhances tolerance to drought and high-salinity. Plant Cell Rep..

[37] Jin L.G., Li H., Liu J.Y. (2010). Molecular characterization of three novel ethylene responsive 2 element binding factor genes from cotton. J. Integr. Plant Biol..

[38] Oñate-Sánchez L., Singh K.B. (2002). Identification of Arabidopsis Ethylene-Responsive Element Binding Factors with Distinct Induction Kinetics after Pathogen Infection. Plant Physiol..

[39] Shinwari Z.K., Nakashima K., Miura S., Kasuga M., Seki M., Yamaguchi-Shinozaki K., Shinozaki K. (1998). An Arabidopsis Gene Family Encoding DRE/CRT Binding Proteins Involved in Low-Temperature-Responsive Gene Expression. Biochem. Biophys. Res. Commun..

[40] Gilmour S.J., Sebolt A.M., Salazar M.P., Everard J.D., Thomashow M.F. (2000). Overexpression of the Arabidopsis CBF3 transcriptional activator mimics multiple biochemical changes associated with cold acclimation. Plant Physiol..

[41] Mizoi J., Shinozaki K., Yamaguchi-Shinozaki K. (2012). AP2/ERF family transcription factors in plant abiotic stress responses. BBA Gene Regul. Mech..

[42] Tang Y., Qin S., Guo Y., Chen Y., Wu G. (2016). Genome-Wide Analysis of the AP2/ERF Gene Family in Physic Nut and Overexpression of the JcERF011 Gene in Rice Increased Its Sensitivity to Salinity Stress. PLoS ONE.

[43] Zhang C.H., Shangguan L.F., Ma R.J., Sun X., Tao R., Guo L., Korir N.K., Yu M.L. (2012). Genome-wide analysis of the AP2/ERF superfamily in peach (Prunus persica). Genet. Mol. Res..

[44] Zhou L.M., Tang Y.X., Wu Y.M. (2012). Genome-Wide Analysis of AP2/ERF Transcription Factor Family in Zea Mays. Curr. Bioinform..

[45] Yao W., Zhang X., Zhou B., Zhao K., Li R., Jiang T. (2017). Expression Pattern of ERF Gene Family under Multiple Abiotic Stresses in Populus simonii × P. nigra. Front. Plant Sci..

[46] Lynch M. (2002). Genomics. Gene duplication and evolution. Science.

[47] Li M.Y., Wang F., Jiang Q., Li R., Ma J. (2013). Genome-wide Analysis of the Distribution of AP2/ERF Transcription Factors Reveals Duplication and Elucidates their Potential Function in Chinese Cabbage (Brassica rapa ssp. pekinensis). Plant Mol. Biol. Rep..

[48] Lata C., Mishra A.K., Muthamilarasan M., Bonthala V.S., Khan Y., Prasad M. (2014). Genome-Wide Investigation and Expression Profiling of AP2/ERF Transcription Factor Superfamily in Foxtail Millet (Setaria italica L.). PLoS ONE.

[49] Lang K., Bi S.D., Li F. (2018). Genome-wide analysis of expansion and contraction of gene families in parasitic wasps. J. Anhui Agric. Univ..

[50] Ken W., Ó’hUigín C. (2016). Significance of positive selection and gene duplication in adaptive evolution: In memory of Austin L. Hughes. Immunogenetics.

[51] Papdi C., Pérez-Salamó I., Joseph M.P., Giuntoli B., Szabados L. (2015). The low oxygen, oxidative and osmotic stress responses synergistically act through the ethylene response factor VII genes RAP2.12, RAP2.2 and RAP2.3. Plant J..

[52] Berrocal-Lobo M., Molina A., Solano R. (2002). Constitutive expression of ETHYLENE-RESPONSE-FACTOR1 in Arabidopsis confers resistance to several necrotrophic fungi. Plant J..

[53] Zhang Z., Wang J., Zhang R., Huang R. (2012). The ethylene response factor AtERF98 enhances tolerance to salt through the transcriptional activation of ascorbic acid synthesis in Arabidopsis. Plant J..

[54] Robert C. (2004). Edgar. MUSCLE: Multiple sequence alignment with high accuracy and high throughput. Nucleic Acids Res..

[55] Fujimoto S.Y., Ohta M., Usui A., Shinshi H., Ohme-Takagi M. (2000). Arabidopsis ethylene-responsive element binding factors act as transcriptional activators or repressors of GCC box-mediated gene expression. Plant Cell.

[56] Lifang H., Shiqiang L. (2011). Genome-wide identification and phylogenetic analysis of the ERF gene family in cucumbers. Genet. Mol. Biol..

[57] Fahad S., Hussain S., Matloob A., Khan F.A., Khaliq A., Saud S., Hassan S., Shan D., Khan F., Ullah N. (2015). Phytohormones and plant responses to salinity stress: A review. Plant Growth Regul..

[58] Xiong L., Zhu J.K. (2010). Molecular and genetic aspects of plant responses to osmotic stress. Plant Cell Environ..

[59] Wang Z., Xiong L., Li W., Zhu J., Zhu J. (2011). The plant cuticle is required for osmotic stress regulation of abscisic acid biosynthesis and osmotic stress tolerance in Arabidopsis. Plant Cell.

[60] Zhou W., Wang Y., Li B., Petijová L., Hu S., Zhang Q., Niu J., Wang D., Wang S., Dong Y. (2020). Whole-genome sequence data of *Hypericum perforatum* and functional characterization of melatonin biosynthesis by N-acetylserotonin O-methyltransferase. J. Pineal Res..

[61] Bailey T.L., Nadya W., Chris M., Li W.W. (2006). MEME: Discovering and analyzing DNA and protein sequence motifs. Nucleic Acids Res..

[62] Chen C., Chen H., Zhang Y., Thomas H.R., Xia R. (2020). TBtools: An Integrative Toolkit Developed for Interactive Analyses of Big Biological Data. Mol. Plant.

[63] Hu B., Jin J., Guo A.Y., Zhang H., Luo J., Gao G. (2015). GSDS 2.0: An upgraded gene feature visualization server. Bioinformatics.

[64] Magali L., Patrice D., Gert T., Marchal K., Moreau Y., Van de Peer Y., Rouzé P., Rombauts S. (2002). PlantCARE, a database of plant cis-acting regulatory elements and a portal to tools for in silico analysis of promoter sequences. Nucleic Acids Res..

[65] Hurst L.D. (2002). The Ka/Ks ratio: Diagnosing the form of sequence evolution. Trends Genet..

[66] Suyama M., Torrents D., Bork P., Suyama M., Torrents D., Bork P. (2006). PAL2NAL: Robust conversion of protein sequence alignments into the corresponding codon alignments. Nucleic Acids Res..

[67] Conesa A., GÖtz S., García-Gómez J.M., Terol J., Talón M., Robles M. (2005). Blast2GO: A universal tool for annotation, visualization and analysis in functional genomics research. Bioinformatics.

[68] Harris M.A., Clark J., Ireland A., Lomax J., Ashburner M., Foulger R., Eilbeck K., Lewis S., Marshall B., Mungall C. (2004). The Gene Ontology (GO) Database and Informatics Resource. Nucleic Acids Res..

[69] Carbon S., Ireland A., Christopher J.M., Shu S., Marshall B., Lewis S., AmiGO, Hub (2009). AmiGO: Online access to ontology and annotation data. Bioinformatics.

[70] Zhou W., Wang S.Q., Yang L., Sun Y., Zhang Q., Li B., Wang B., Li L., Wang D., Wang Z. (2019). Reference genes for qRT-PCR normalisation in different tissues, developmental stages, and stress conditions of Hypericum perforatum. PeerJ.

[71] Zhou W., Zhang Q., Sun Y., Yang L., Wang Z. (2020). Genome-wide identification and characterization of R2R3-MYB family in Hypericum perforatum under diverse abiotic stresses. Int. J. Biol. Macromol..

