# Supplementary material for: Genome-Wide Analysis and the Expression Pattern of the ERF Gene Family in Hypericum perforatum
Source: Plants (Basel). 2021 Jan 11;10(1):133. doi: 10.3390/plants10010133 (PMC7827068; doi:10.3390/plants10010133)
Supplement: Supplementary file 1 [file plants-10-00133-s001.zip › Supplementary data for xml checked by authors/plants-1028247-Supplementary for xml-checked by authors.docx]

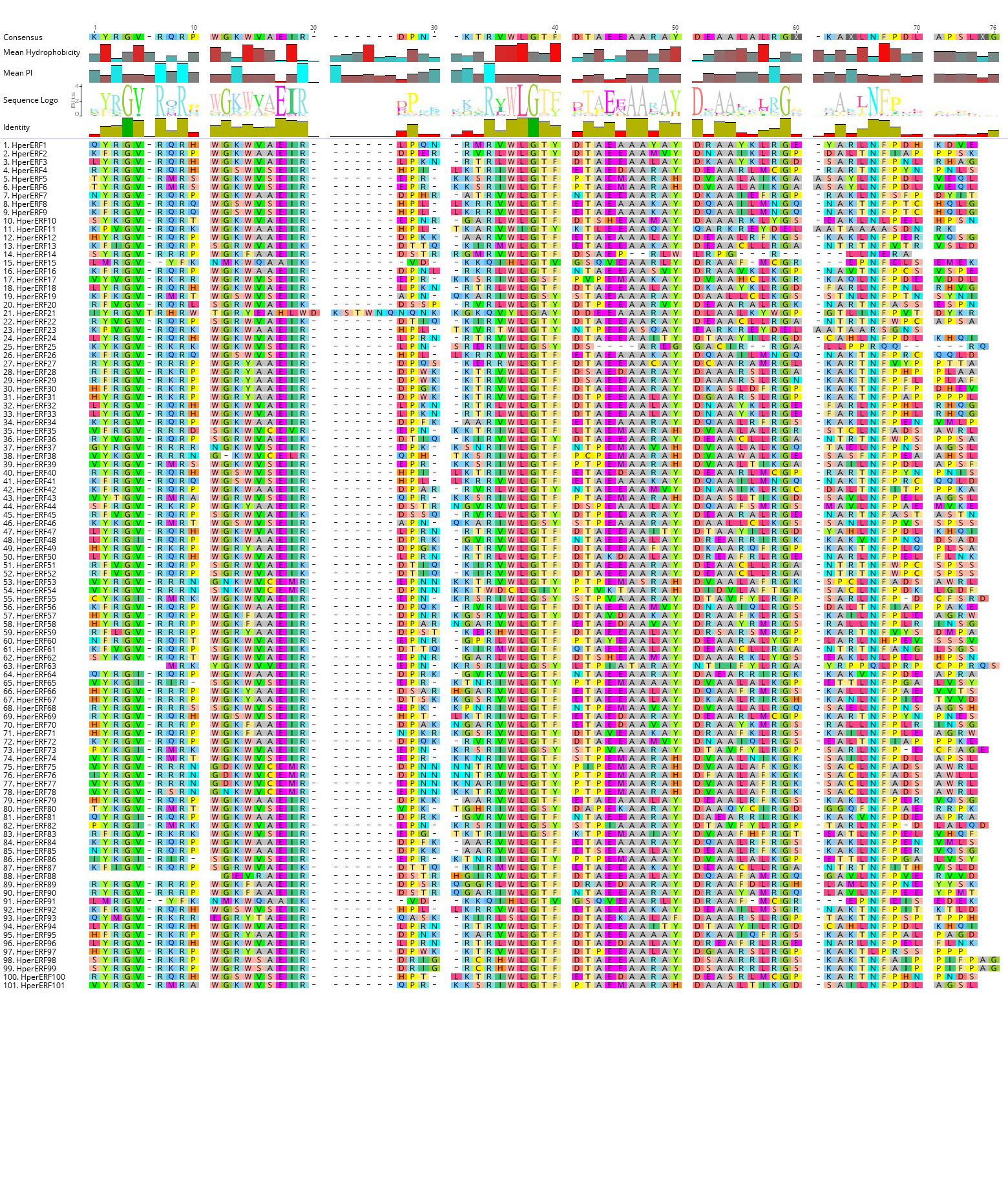


**Figure S1.** The multiple alignment analysis of the AP2/ERF domains from the 101 *H. perforatum* ERF proteins.


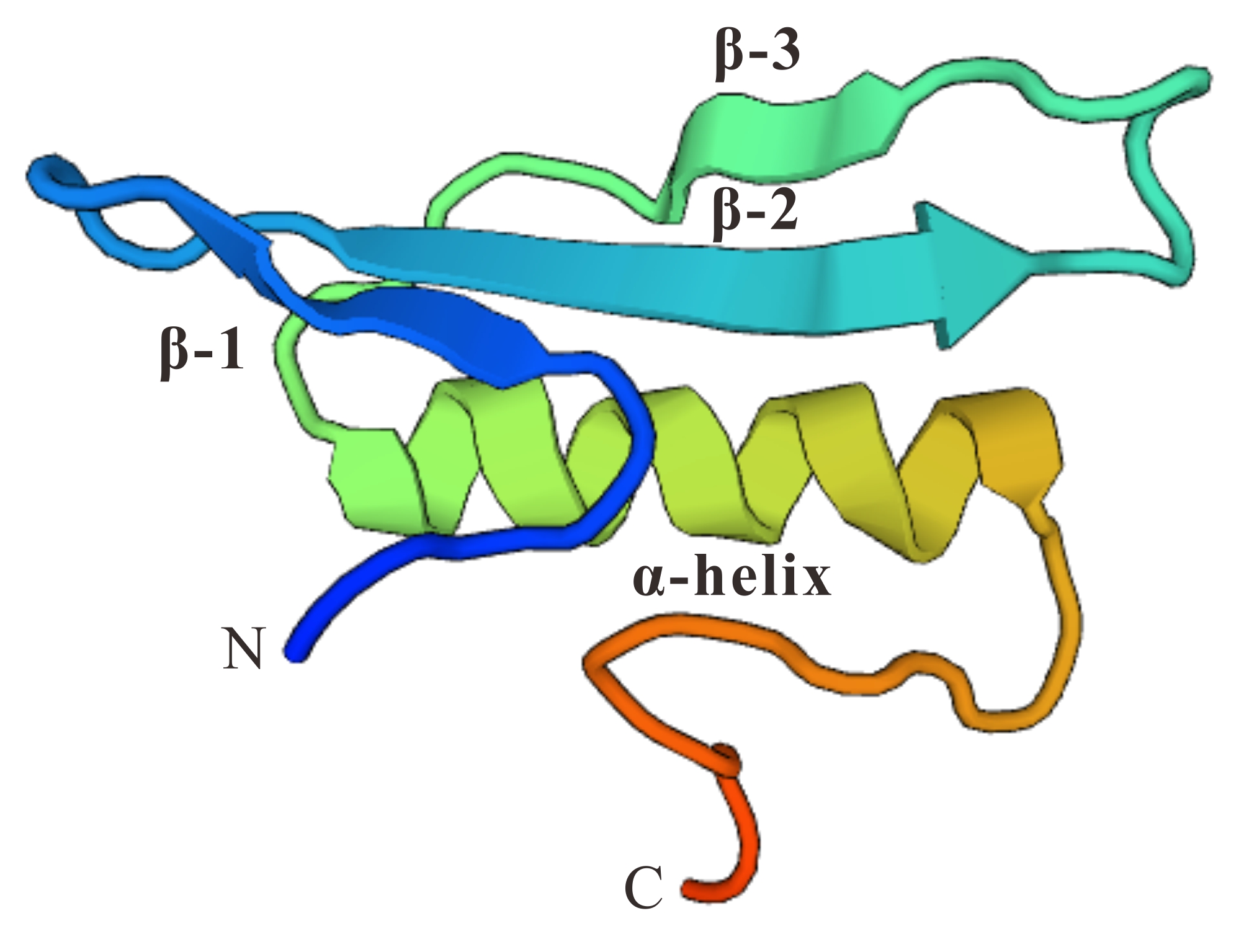


**Figure S2.** The 3D structure of the AP2/ERF domain. The secondary structural elements of this domain, including α-helix and β-sheet, are labeled and the ends are N-terminal and C- terminal, respectively.


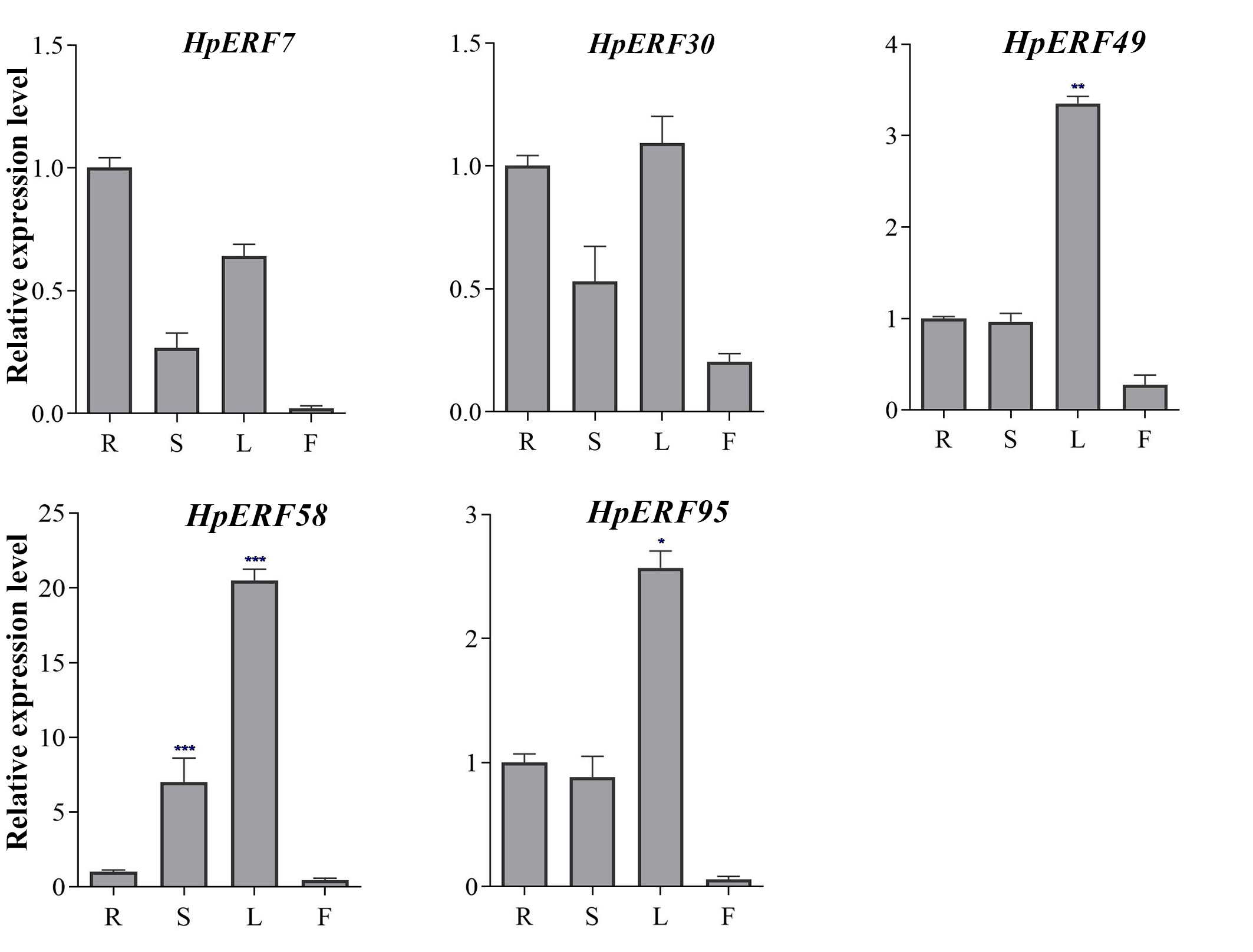


**Figure S3.** The expression patterns of five *HpERFs* in the root (R), stem (S), leaf (L), and flower (F) tissues examined by qPCR. Asterisks (*p* < 0.05) indicate significant differences compared with the control (* *p* < 0.05, ** *p* < 0.01, *** *p* < 0.001).
